# Biobanked Glioblastoma Patient-Derived Organoids as a Precision Medicine Model to Study Inhibition of Invasion

**DOI:** 10.3390/ijms221910720

**Published:** 2021-10-03

**Authors:** Emilie Darrigues, Edward H. Zhao, Annick De Loose, Madison P. Lee, Michael J. Borrelli, Robert L. Eoff, Deni S. Galileo, Narsimha R. Penthala, Peter A. Crooks, Analiz Rodriguez

**Affiliations:** 1 Department of Neurosurgery, Winthrop P. Rockefeller Cancer Institute, University of Arkansas for Medical Sciences, Little Rock, AR 72205, USA; edarrigues@uams.edu (E.D.); ehzhao@uark.edu (E.H.Z.); adeloose@uams.edu (A.D.L.); mpLee@uams.edu (M.P.L.); 2 Department of Radiology, University of Arkansas for Medical Sciences, Little Rock, AR 72205, USA; mjborrelli@uams.edu; 3 Department of Biochemistry and Molecular Biology, University of Arkansas for Medical Sciences, Little Rock, AR 72205, USA; RLEoff@uams.edu; 4 Department of Biological Sciences, University of Delaware, Newark, DE 19716, USA; dgalileo@udel.edu; 5 Department of Pharmaceutical Sciences, College of Pharmacy, University of Arkansas for Medical Sciences, Little Rock, AR 72205, USA; NRPenthala@uams.edu (N.R.P.); pacrooks@uams.edu (P.A.C.)

**Keywords:** glioblastoma, organoids, invasion, patient-derived, compound, drug screening, precision medicine

## Abstract

Glioblastoma (GBM) is highly resistant to treatment and invasion into the surrounding brain is a cancer hallmark that leads to recurrence despite surgical resection. With the emergence of precision medicine, patient-derived 3D systems are considered potentially robust GBM preclinical models. In this study, we screened a library of 22 anti-invasive compounds (i.e., NF-kB, GSK-3-B, COX-2, and tubulin inhibitors) using glioblastoma U-251 MG cell spheroids. We evaluated toxicity and invasion inhibition using a 3D Matrigel invasion assay. We next selected three compounds that inhibited invasion and screened them in patient-derived glioblastoma organoids (GBOs). We developed a platform using available macros for FIJI/ImageJ to quantify invasion from the outer margin of organoids. Our data demonstrated that a high-throughput invasion screening can be done using both an established cell line and patient-derived 3D model systems. Tubulin inhibitor compounds had the best efficacy with U-251 MG cells, however, in ex vivo patient organoids the results were highly variable. Our results indicate that the efficacy of compounds is highly related to patient intra and inter-tumor heterogeneity. These results indicate that such models can be used to evaluate personal oncology therapeutic strategies.

## 1. Introduction

Glioblastoma (GBM), also known as grade IV astrocytoma, is the most common malignant primary brain tumor in adults. GBM has both intra- and inter-tumor heterogeneity and possesses a variety of genetic and epigenetic mutations that drive tumor cell motility to cause cells to invade, infiltrate, and colonize close and distant surrounding normal brain tissue [1]. Due to the aggressive nature of invasion in GBM, complete surgical resection is not feasible, and remaining cancer cells can undergo mutations to survive ionizing-radiotherapy and chemotherapy (e.g., DNA-alkylating agent temozolomide (TMZ)), leading to recurrence [2]. The GBM median survival time, even after surgery, radiation, and chemotherapy, is 15 months after the initial diagnosis [3,4]. GBM also occurs in a complex tumor microenvironment (TME) made of extracellular matrix (ECM) components and infiltrated cancer-associated cells such as immune cells, several types of stromal and endothelial cells, and glial cells that crosstalk with the cancer cells, and this plays a critical role in tumor invasion [4,5,6,7].

A critical factor limiting the progress of GBM therapeutic innovation is the lack of simple and reliable in vitro models for testing therapeutic strategies. Current standard models are typically 2-dimensional (2D) cell culture systems which do not mimic the genetic and microenvironmental complexity of GBM [8]. Some of the best options available today are in vivo models such as patient-derived xenografts (PDX). However, long engraftment periods and high costs make PDX models limiting for many research groups [9]. Advancements in cell culture techniques have seen the rapid evolution of more complex in vitro cell culture models that include three-dimensional (3D) multicellular spheroids, organoids, bio-printing, and organ-on-a-chip models. These models have been shown to have improved characteristics in comparison to 2D models which include abundant extracellular matrix (ECM), cell heterogeneity and signaling variation; hypoxic, nutrition, and pH gradients; and higher therapeutic resistance and mutations similar to the parent tumor, which thus make them more suitable for cancer therapy research [10,11,12,13,14].

**Figure 1:**
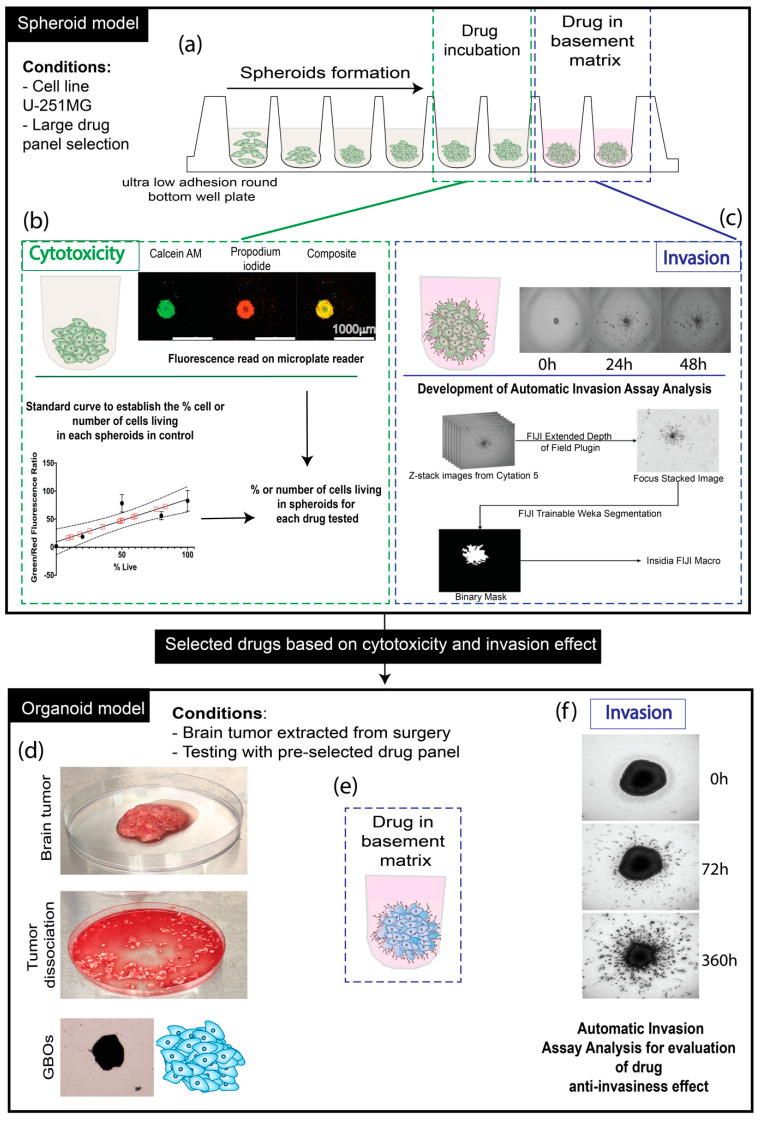
Schematic of experimentation. (**a**) U-251 MG cell spheroid formation in an ultra-low attachment round bottom well plate, followed by either drug incubation for cytotoxicity analysis or drug (10 µM) distributed in the basement membrane matrix Matrigel for invasion assay analysis. (**b**) Cytotoxicity assessment of the compounds on spheroids by interpolation of the standard curve established based on Calcein AM (green: living cells) and propidium iodide (red: dead cells) fluorescence intensity related to the known ratio of living/dead cells (0, 20, 50, 80, and 100%), with the establishment of a standard curve allowing us to interpolate (following mathematical model with Graphpad: Prism) (Figure S2) the percentage of living cells and also the number of cells related to the fluorescence intensity. (**c**) Development of an automatic invasion assay analysis using FIJI/ImageJ and INSIDIA macro. (**d**) Brain tumor extracted from surgery, followed by dissection and dissociation to constitute the GBOs. (**e**) Transfer of the GBOs in an ultra-low adhesion well plate with the drug incorporated in the basement matrix. (**f**) Evaluation of the invasiveness of the GBOs for 360 h maximum, using automatic invasion assay analysis.

Due to the invasiveness of GBM, 3D models reproducing this feature are essential, especially when studying, selecting, and screening a large group of compounds that inhibit invasion. In this study, using multicellular spheroids developed with the U-251 MG cell line, which is well-characterized [15], and has been used in the National Cancer Institute’s published A Large Matrix of AntiNeoplastic Agent Combinations (NCI-ALMANAC) [16], we screened our library of anti-invasive compounds, composed of NF-kB, GSK-3-B, COX-2, and tubulin inhibitors by developing and testing an automatic quantitative Matrigel-based invasion assay. Since GBM cancer cells spread in the brain primarily along the perivascular spaces between the endothelial cells and astrocytic end feet which comprise the blood brain barrier [17], we used a Matrigel invasion assay. The major component of Matrigel is laminin, which is a major component of the basement membranes surrounding blood vessels [18,19]. Developing a GBM 3D model that also maintains a characteristic TME is a challenging hurdle. To answer this need, we decided to utilize patient-derived glioblastoma organoid cultures (GBOs). This model has been shown to preserve tumor heterogeneity and complexity, to recapitulate inter-and intra-tumoral heterogeneity, and to maintain many key features of GBM, such as its cytoarchitecture and cell-cell interactions [20,21,22]. Our goal was to validate the use of GBOs in 3D pre-clinical models for precision medicine, specifically assaying invasiveness using our automated Matrigel-based invasion assay [23]. We used patient-derived tissue obtained from surgery and our precision medicine biobank [24]. Our study confirms that compound efficacy is highly variable between GBOs from different patients, indicating that precision medicine models such as ours may result in improved therapeutic solutions for patients (Figure 1). Our pilot GBO studies indicate the need for the further development of high-throughput screening processes with patient-derived tissue.

## 2. Results

### 2.1. Cytotoxicity of the Compounds on U-251 MG Spheroids

**Figure 2:**
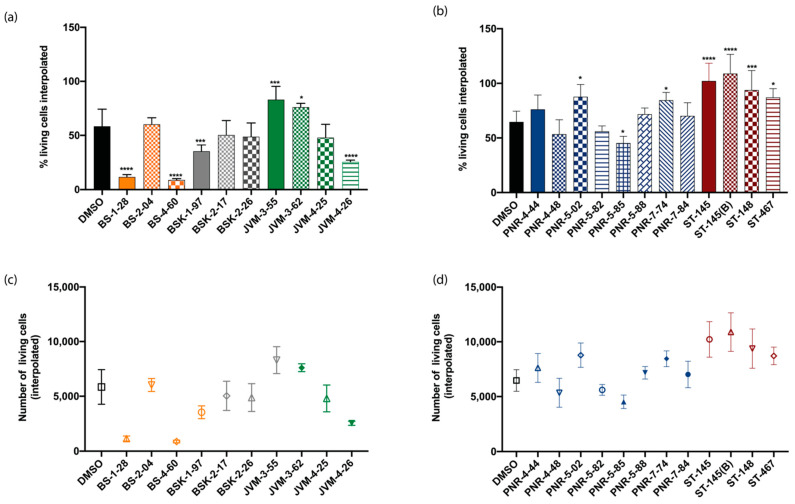
Determination of cytotoxicity on a U-251 MG cell 3-D model with percentage of living cells interpolated for (**a**) the first series of compounds tested: BS, BSK, and JVM, and for (**b**) the second series of compound tested: PNR and ST. Respective numbers of living cells interpolated for (**c**) the first series of compounds tested: BS, BSK, and JVM, and for (**d**) the second series of compounds tested: PNR and ST. Statistical analysis for significance determination was done using one-way ANOVA with the control (DMSO) as the comparative model with the Dunnett model, ns (non -significant) for adjusted *p* > 0.05,* for adjusted *p* ≤ 0.05, *** *p* ≤ 0.001, and **** *p* ≤ 0.0001. For each experiment, n = 3.

U-251 MG is an established GBM cell line [15], part of the NCI-60 human cancer cell lines [25], with the ability to form spheroids with invasive properties [26]; it has been used in the screening of pharmaceutical agents to identify, characterize, and select novel compounds with growth inhibition and/or killing of tumor cells. A series of 22 different compounds called BS, BSK, JVM PNR, and ST [27,28,29,30,31,32,33,34] whose the chemical structures, details, and activity are explained in Table 1 and Figure S1, were incubated with the spheroids for 48 h to define the toxicity the effects on their invasiveness. These compounds have anti-invasive properties, as they interfere with pathways of cell invasion (Figure 2). The goal of our initial screening was to identify compounds that were able to stop the invasion independently of their induced toxicity. We selected a fixed concentration of 10 µM for all compounds based on preliminary data (not shown) to support the selection of compounds to validate the GBO model.

Our results indicated three categories of compounds in terms of viability, organized in function of their statistical relevance: (1) with cytotoxic effect or higher death than the control (5/22 compounds), such as BS-1-28, BS-4-60, and JVM-4-26, which had the most cytotoxic effect; (2) neutral, with no difference compared to the control (9/22 compounds); and (3) with higher viability than the control (8/22 compounds), such as JVM-3-55, PNR-5-02, ST-145 (Figure 2a,b; Table 2).

For the concentration tested, the correlation of these three groups in function of their cytotoxic effects and their activity (Table 2) indicates that, for most compounds, NFk-B inhibitors were the ones with the most cytotoxicity. Given that NFk-B signaling pathways are typically overexpressed in cancers to direct growth and progression, it is not surprising that the inhibition of these pathways would lead to cytotoxicity [35]. By contrast, most of the tubulin inhibitors were non-toxic or even supported the growth of the cells.

### 2.2. Invasion Inhibition of the Compounds on U-251 MG Cell Spheroids and the Development and Implementation of Automatic Invasion Assay Analysis

**Figure 3:**
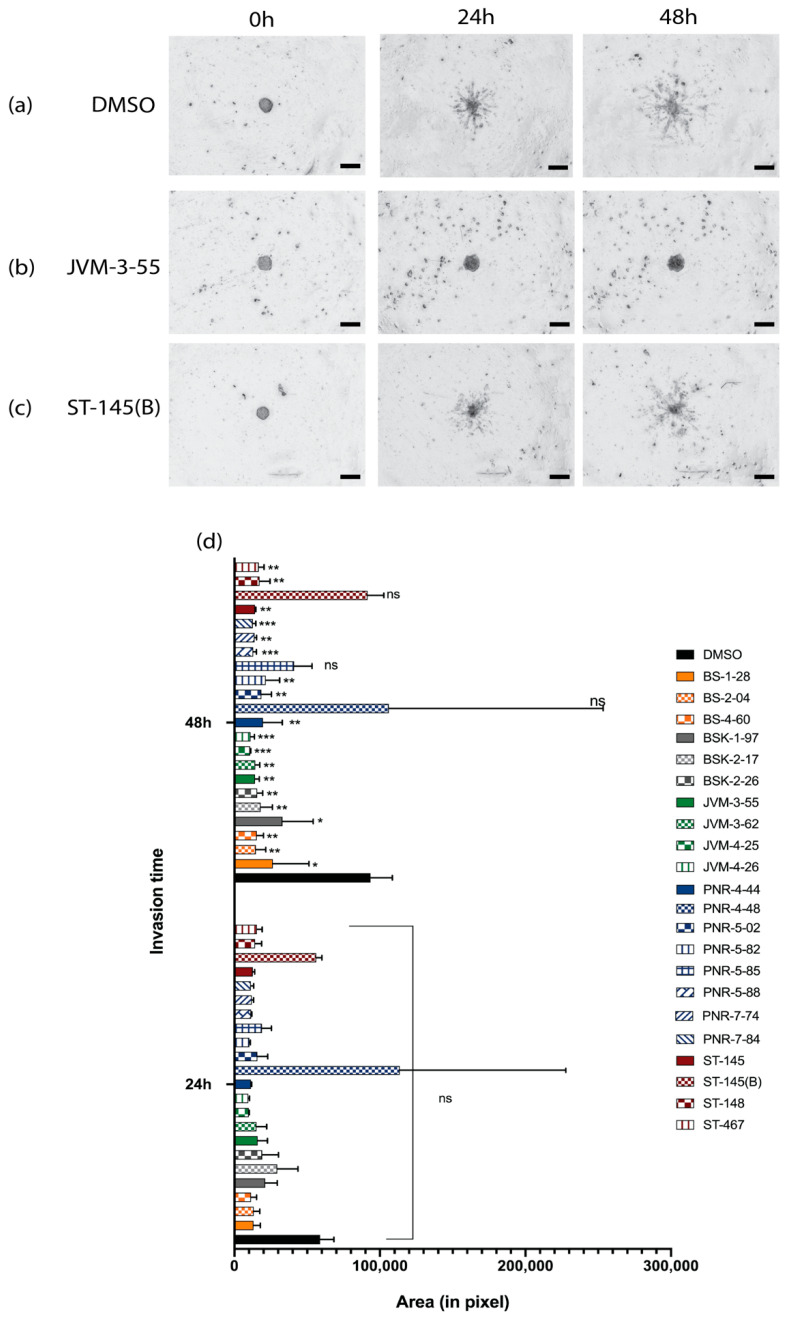
Evaluation of invasion ability and implementation of an automatic invasion analysis assay. Shown are brightfield images recorded on Cytation 5, using a 4× objective lens, overlayed images from z-stack of embedded spheroids in a basement membrane matrix for 0, 24, and 48 h for (**a**) DMSO, (**b**) JVM-3-55, and (**c**) ST-1459(B). Spheroids are large dark bodies in the center of images, and invading cells are smaller dark protrusions from the central spheroid core. (**d**) Quantitation of invasion was determined by the automatic measurement of the area of cells that invaded the matrix (in pixels) for the different times of incubation (0, 24, and 48 h). Statistical analysis of significance was done using two-way ANOVA with the control (DMSO) for the different times of invasion control as the comparative model with the Dunnett model, ns (non-significant) for adjusted *p* > 0.05, * for adjusted *p* ≤ 0.05, ** for adjusted *p* ≤ 0.01, *** *p* ≤ 0.001 For each experiment, n = 3. Scale bar: 200 μm, brightfield images were recorded on Cytation 5 using a 4× objective lens.

A 3D tumor spheroid-based functional assay to model invasion was accomplished by seeding spheroids in a basement membrane matrix (Matrigel^®^, Bedford, MA, USA) containing a fixed and defined concentration of the different compounds. Quantification of the invasion was done using time-series image capture (0, 24, and 48 h) in z-stack using the macro INSIDIA (INvasion SpheroID ImageJ Analysis) [36], and an extended depth-of-field algorithm [37] developed for FIJI/ImageJ [38]. The 3D spheroids were able to invade the basement matrix by creating multiple multicellular protrusions, confirming that this 3D model could be used to estimate and screen different compounds for invasion inhibition (Figure 3a–c). As a first observation, in DMSO, the invasion could be accessed and was visible after 24 h. Invasion also was visible after 24 h for most of the compounds, but inhibition of invasion by compounds became statistically significant after 48 h for most of the compounds tested (Figure 3d).

During that time frame, our data showed that very few compounds impaired the invasion of the cells (only 3/22 tested; two tubulin inhibitor compounds (ST-145(B) and PNR-4-44), and one COX-2 inhibitor (PNR-5-82) compound). For these compounds, spheroids remained rounded (e.g., Figure 3b). When expansion was recorded, most compounds were able to reduce by half the protrusion invasion compared to the control. The tubulin inhibitors were the ones that showed the most effectiveness compared to the control, and inhibited the invasion of the spheroids in the basement matrix.

Cytotoxic effects and invasion ability are summarized in Table 2. The results are organized in three sections based on their classification within the column “Cytotoxicity effect in comparison to control,” with (-) indicating higher cytotoxicity or death induced compared to the control, (=) indicating no difference compared to the control, and (+) indicating higher viability than the control. For each section, invasion was then defined as either low, moderate, or high in a function of the area of invasion measured (Figure 3). Interestingly, toxicity and invasion inhibition did not correlate. Compounds that induced cell death sometimes still had high-to-moderate invasion, especially for the NFk-B inhibitors which, despite their higher toxicity, allowed a high-to-moderate invasion. In contrast, the tubulin inhibitors which do not show any significant toxicity, and even favor the proliferation of cells, were what controlled most of the invasion. Based on these results, we decided to select compounds with no toxicity like PNR-5-88 (COX-2 inhibitor) and PNR-7-84 (Tubulin inhibitor) for GBOs screening, with respectively moderate- to very high invasion inhibition effects. We also selected JVM-3-55 (NFk-B) due to the lack of toxicity (despite this group showing in majority a cytotoxic effect) combined with invasiveness inhibition.

### 2.3. Development of GBOs

**Figure 4:**
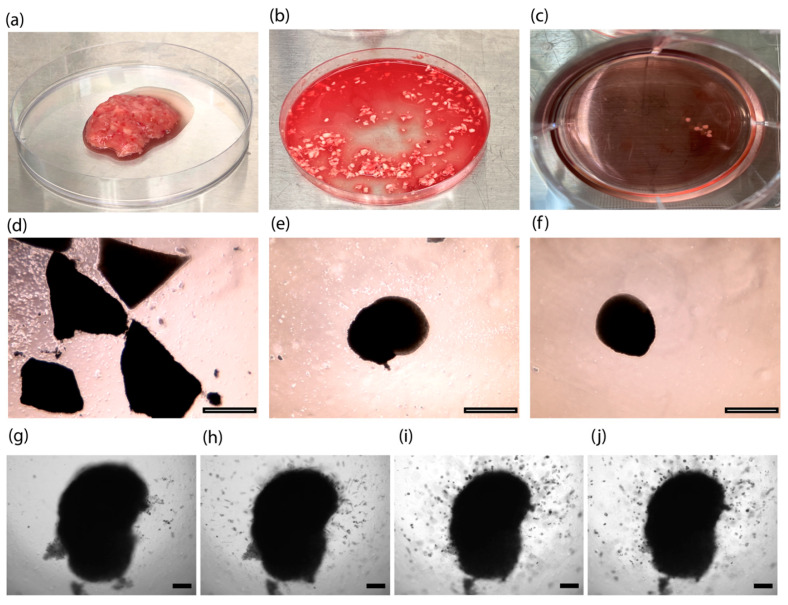
Development of GBOs from: (**a**) a tumor resected in the operating room coming as a bulk tumor, or (**b**) already dissociated in small pieces with a surgical blade until reaching less than 500 µm size. (**c**) Small pieces of tumor are forming GBOs after 15 days in the incubator. (**d**) GBOs are cut into small fragment pieces and incubated in media at t = 0. (**e**) GBOs after t = 7 days in culture media and under permanent shaking. (**f**) GBOs after t = 15 days in culture media and under permanent shaking. GBOs embedded in a matrix to verify invasion properties for (**g**) 0 h, (**h**) 24 h, (**i**) 120 h, and (**j**) 168 h. Scale bar from (**d**) to (**f**): 1000 μm, obtained with x2.5 objective lens, under Evos microscope. Scale bar from (**g**) to (**j**): 200 μm, brightfield images were recorded on Cytation 5, using a 4× objective lens.

Our GBOs are a type of organoid that was made not by dissociating tumors, but by mincing tumors into small pieces (<1 mm) (Figure 4b,d) and culturing them under continuous shaking to favor organoid formation, nutrient diffusion, and gas (oxygen) exchange, with serum-free conditions or in the presence of the basement membrane matrix [21,22]. To develop GBOs, we obtained fresh surgically resected GBM tumor tissue from consented patients, which were biobanked and deidentified under Institutional Review Board (IRB) protocol (#228443). In collaboration with our neurosurgical team [24], we confirmed that our surgical specimens used for generation of GBOs did not have necrosis (Figure 4a). In this study, GBOs were maintained for 15 days (Figure 4c), during which they generally became rounded (Figure 4e–f), as observed in previous studies [21,22]. After 15 days, one patient’s GBOs were seeded in Matrigel (without DMSO or compound) to validate their invasive ability. As with the U-251 MG cell spheroid model, protrusions were visible after 24 h of incubation.

### 2.4. Determination of Invasion Inhibition on GBOs by Selected Compounds

**Figure 5:**
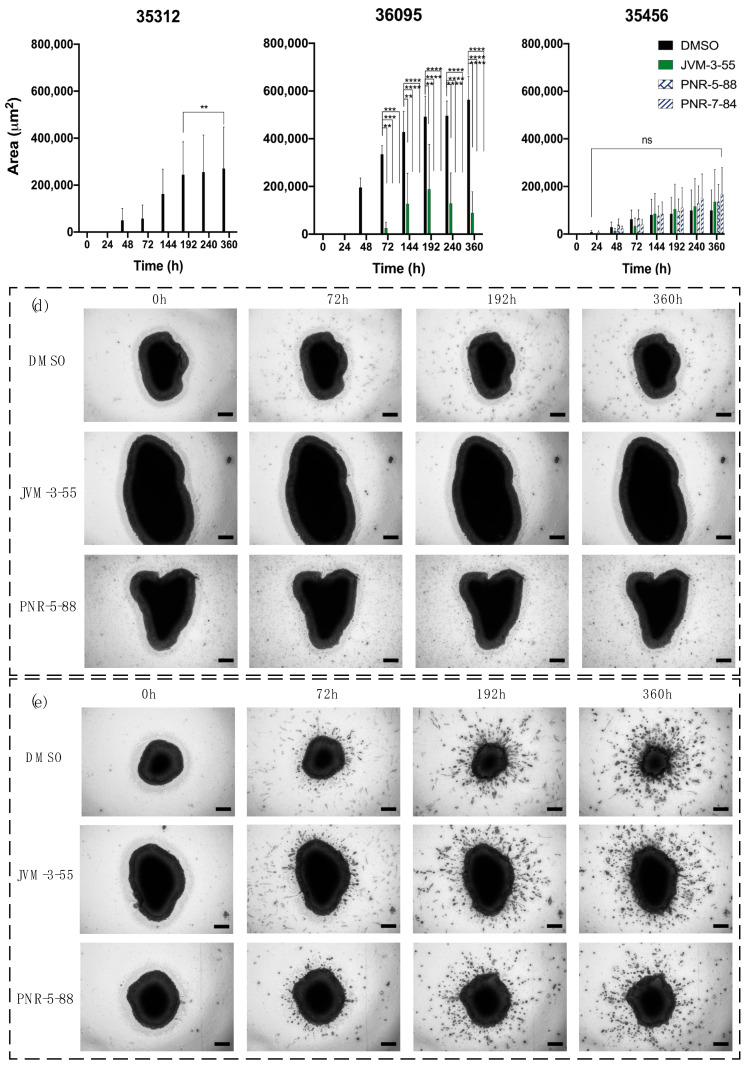
Evaluation of invasion ability of selected compounds on GBOs from different patients. The invasion was determined by automatic measurement of the area of cells invading into the matrix (μm^2^) in a function of the different times of incubation for patients (**a**) 35312, (**b**) 36095, and (**c**) 35456. The statistical analysis for significance was done using two-way ANOVA with the control (DMSO) for the different times of invasion as the comparative model with the Dunnett model, ns (non -significant) for adjusted *p* > 0.05,** for adjusted *p* ≤ 0.01, *** *p* ≤ 0.001, and **** *p* ≤ 0.0001. For each experiment, n = 3. Brightfield images were recorded on Cytation 5 using a 4× objective lens, in z-stacks of embedded GBOs in the matrix for 0, 72, 192, and 360 h with either DMSO, JVM-3-55, or PNR-5-88 for patients 36095 (**d**) and 35456 (**e**). Scale bar: 200 μm.

We processed different GBM organoids and selected 3 patients, referenced as follows: 35312, 36095, and 35456. After 15 days, triplicate GBOs from each patient were seeded with either DMSO (control), JVM-3-55, PNR-5-88, or PNR-7-84, and embedded in Matrigel for 0, 24, 48, 72, 144, 192, 240, and 360 h. An automatic invasion assay protocol was applied to determine the resulting invasion area outward from the GBOs and any potential inhibition of invasion by the compounds (Figure 5). For patient 35312 GBOs, inhibition of invasion was observed using all compounds and became significant after 192 h. For patient 36095 GBOs, inhibition of invasion was visible after 72 h for all three compounds. However, JVM-3-55 was less inhibitive than PNR-5-88 or PNR-7-84. The imaging showed rounded GBOs from this patient without the presence of invasive protrusions, except when in DMSO. For patient 35456, none of the compounds inhibited the invasion from the GBOs. The imaging indicated a large presence of protrusions after about 72 h. Thus, cells from different patients with the same GBM diagnosis exhibited different results to invasion inhibitors.

## 3. Discussion and Conclusions

Glioblastoma, due to their complex TME and a wide range of mutations, can invade, infiltrate, and colonize close- and distant surrounding healthy brain tissue. Some current in vitro models fail to mimic these in vivo characteristics, which delays the discovery and development of small-molecule cancer inhibitors. The goal of this study was to evaluate a panel of compounds which were already tested and screened in conventional 2D culture and animal models in a patient-derived glioblastoma organoids model for their efficacy in stopping the invasive properties of GBM cells. The ability to use this model to screen compounds in 15 days allows for the potential to support clinical trials with the benefit of evaluating new drugs that are the most effective for individual patients, developing precision medicine as a new technique for the clinical management of GBM.

First, a GBM 3D culture as spheroids or organoids made from commercial cell lines or = patient-derived cancer cells was used to investigate drug response and mechanisms of resistance [39,40] and radioresistance [41]. Spheroids have also been shown to mimic invasiveness when implanted on an external basement membrane matrix, such as Matrigel [42], but have been rarely studied in the case of GBM. One of the reasons is the difficultly of processing a large number of recorded images using open-access software to quantify the three-dimensional invasion of cells through the matrix. Using FIJI/ImageJ, INSIDIA [36], and a new macro to enhance the depth of the imaging, we have been able to determine the size of the area invading the surrounding core of the spheroids, allowing for the screening of a large number of compounds. Here, we have shown, by evaluating cytotoxicity through an established standard curve, and invasiveness through the development of an automatic invasion assay analysis, that the GBM spheroid model has an ability of invasion in a basement membrane matrix for the screening of many compounds. As a proof of concept, we studied the U-251 MG GBM cell line to develop the algorithm by using a fixed concentration of the different compounds.

Our results recorded with the implementation of the algorithm encourage future work on a larger number of GBM cell lines and, more specifically (1) GBM cell lines that are part of the NCI-60 cell lines, and (2) patient-derived cells dissociated from GBM tumors. We believe that the screening of compounds based on their cytotoxicity should also be correlated with invasion data. This study highlights the evidence that toxicity can be induced but invasion is still unaffected. Due to the diversity of compounds and their mechanisms of action, as well as the diversity of GBM, the standardization of methods with spheroids that are highly repeatable and easy to form, culture, and treat will be necessary to identify specific genetic biological mutations that can be treated with specific types of compounds to assess their IC_50_ and pharmacokinetics.

Despite the ability of spheroids to invade a basement membrane matrix, it is recognized that spheroids lack cellular heterogeneity because they are made of only cancer cells. Invasiveness is linked to cancer–TME crosstalk, and therefore more advanced 3D models that replicate the complex TME are likely the ideal systems to study invasion [43,44,45]. Developing a GBM 3D model that also possesses a surrounding normal human brain microenvironment is another challenging hurdle. The emergence of human brain organoids or organotypic models combining patient-specific GBMs that use both patient-derived glioma stem cells (GSCs) and human stem cells looks to be very promising. Unlike animal brains, which are used in PDX models, human brain organoids providing a species-specific microenvironment show invasive protrusions and microtube networks [46,47]. The advantages of such models will be their large scalability, reproducibility, and standardization. These types of 3D cultures developed from stem cells reproducing the phenocopy of patients are extremely promising. However, they are costly in time (>30 days in general). GBOs are a unique 3D model obtained from resected tumors with minimal processing and the absence of manual or chemical dissociation, preserving tumor integrity and TME for at least 2 weeks under in vitro culture.

Following recent achievements in the discovery of GBOs [22,23], we studied this new model to evaluate its effectiveness on invasiveness of drugs and small-molecule inhibitors that have been effective on motility in 2D cultures [48], or invasiveness in simpler 3D spheroid models, yet may not appear to be effective for patients in all cases or in clinical trials. The rapid evaluation of compounds on the invasiveness of individual patient’s cells in the more heterogeneous and tissue-like context of the GBO model may reveal that such compounds are effective for some patient’s cells and not others, thus further helping to guide a personalized therapeutic approach. Potential reasons for this could be: (1) the resected tumors possess different invasiveness, being more or less aggressive in a function of the patient; (2) the part of the tumor resected to make the GBOs was not in an invasive niche of the tumor; (3) patients underwent different treatments and had some GBM recurrence after radiation or chemotherapy, making it possible that the invasion properties of the GBM changed with treatment; (4) the mutations in GBM differed between the different patients.

A limitation of our pilot study with GBOs is that cytotoxicity was not assessed during the drug screens. We were limited by the quantity of surgically resected tissue available and the difficulty in imaging dense 3D structures in Matrigel. In future studies, we will assay cytotoxicity contemporaneously with invasion while in Matrigel, and we are currently working on optimizing these protocols.

Our study demonstrates that GBOs may be an attractive option to consider for assaying invasion inhibition. This study was a pilot to assess the feasibility of this assay, as GBO-based invasion has yet to be published to our knowledge. Another primary limitation that we faced is controlling for the initial size of the GBOs. We are currently evaluating automated systems which can decrease tissue handling time and allow for high throughput scalability and size control. Previous studies that utilized 3D cancer spheroids have demonstrated that size is related to drug diffusivity/penetration [49,50]. However, GBOs are more complex, given the presence of various cell types and stroma. In the future, we will determine the relationship between GBOs size and drug diffusion. To quantify diffusion, fluorescently labeled drugs can be used in combination with two-photon microscopy which can be used to image 3D models with high solidity [51]. We are currently developing these methods to expand our model and address this current key limitation. Ultimately, our goal is to begin the transition from this preclinical model to clinical implementation as a functional precision medicine assay. A main advantage of the GBO model is its ability to replicate the heterogenous microenvironment of the parent tumor [21,22]. Patient-derived models using GBM surgical tissue have been implemented to assess drug efficacy and guide clinical management [52]. To perform a large drug screening pipeline, the GBO model requires more tissue than models in which the tumor is dissociated into cancer cells. GBOs can be potentially ideal for smaller targeted drug panels than spheroids, as they are able to be used in a relatively short time-period to allow integration with a patient treatment scheme.

In conclusion, these results showed that a spheroid-and-GBO model combination can be used to determine the invasion-inhibitive effects of a larger number of compounds, and that our assay can quickly evaluate invasion with the use of an open-access plug-in implemented in FIJI/ImageJ. Due to the inherent variability of GBOs given the heterogenous nature of GBM, in future studies we advocate for the development of a high-throughput system that can undergo both quantitative and qualitative analyses for drug screening. These tools will support the scalability of drug screening in terms of numbers of compounds, time of analysis (lower than the time of surgery recovery), numbers of patients, and quantity of samples that can be used to investigate a new clinical trial set-up as well as implementing a more personalized therapeutic approach.

## 4. Materials and Methods

### 4.1. Drug Panel Selected for Experimentation

Please see as Table 2.

### 4.2. Spheroid Culture, Standard Curve Generation, and Cellular Viability in 3D GBM Spheroids

U-251 MG cells (Sigma-Aldrich, St. Louis, MO, USA, #09063001) were cultured in Dulbecco’s modified Eagle’s medium (DMEM; Fisher Scientific, Hampton, NH, USA) supplemented with 10% Fetal Bovine Serum (FBS), 3.2% nonessential amino acids (NEEA), 4 mmol/L L-Glutamine, and 100 U/mL penicillin, 100 U/mL streptomycin. All supplements were from Fisher Scientific. Spheroids were cultured at 37 °C and 5% CO_2_. U-251 MG cell spheroids were grown by plating 5000 viable cells/well/100 μL using ultra-low attachment round-bottom microplates (Corning, Bedford, MA, USA, #4515) at 37 °C and 5% CO_2_ in DMEM supplemented with 10% FBS, 1% nonessential amino acids (NEEA), 4 mmol/L L-Glutamine, and 100 U/mL penicillin, 100 U/mL streptomycin. Spheroids were grown for 48 h, then treated with 10 µM final concentration of compound for an additional 48 h. They were stained live with Calcein AM/Propidium Iodide (Invitrogen, Waltham, MA, USA). Total fluorescence was read after 30 min, or spheroids were imaged after 20 min using Cytation 5 (Biotek, Winooski, VT, USA). To generate standard viability curves, U-251 MG cells were grown in DMEM supplemented with 10% FBS, 1% nonessential amino acids (NEEA), 4 mmol/L L-Glutamine, and 100 U/mL penicillin, 100 U/mL streptomycin with a total of 10,000 cells per well at specific percentages of living cells (0, 20, 50, 80, and 100%) mixed with specific percentages of dead cells (100, 80, 50, 20, and 0%) to equal 100%. Then, the wells were stained with Calcein AM/Propidium Iodide (Invitrogen). Total fluorescence in each well was read after 30 min using Cytation 5.

### 4.3. Spheroid Culture for Invasion Assay

U-251 MG cell spheroids were grown by plating 5000 viable cells/well/100 μL using ultra-low attachment round bottom microplates for 48 h, as above. After cooling the plate for at least 30 min on ice, 100 µL of media was removed, and the spheroids were treated with 10 µM of compound and 33% of Matrigel^®^ (Corning #351234) final concentration. Migration was observed at 0, 24, and 48 h using Cytation 5.

### 4.4. Spheroid Measurements for Automatic Invasion Assay Analysis

Spheroids treated with various compounds were imaged using brightfield microscopy at 0, 24, and 48 h after treatment with a Cytation 5 Cell Imaging Multi-Mode Reader. Image data for each spheroid consisted of 22 optical sections at different levels along the *z*-axis to create a z-stack at each time point. Images were then processed, segmented, and measured using the FIJI distribution of ImageJ [38].

Each z-stack of 22 optical sections had to be overlayed to form a single projection prior to segmentation. The z-projections produced by Cytation 5 left spheroids out of focus and were thus unsuitable for segmentation. To obtain an in-focus projection of each spheroid, we used an ImageJ implementation of an extended depth-of-field algorithm to fuse each stack into a single focus-stacked image [37]. Shade-off artifacts were then removed from the resulting images using background subtraction in ImageJ.

Images were then segmented using the Trainable Weka Segmentation plugin included with FIJI (trainable segmentation v3.1.2). [53,54] A classifier model was trained using 19 manually selected images representative of the different tumor spheroid morphologies as well as types of extraneous debris found in the image data set. Image features were assigned to one of two classes—“Spheroid” and “Background.” Tumor cells were assigned to the “Spheroid” class, while extraneous debris was assigned to the “Background” class. The resulting model was then used to classify the entire image data set and generate binary masks outlining each tumor spheroid. The binary masks were then run through the INSIDIA FIJI macro [36] to obtain two-dimensional tumor area measurements.

### 4.5. Collection, Dissection, and Processing of Patient Glioblastoma

Fresh surgically resected glioblastoma tissue was placed in sterile phosphate-buffered saline and taken immediately to the lab. The tissue was distributed and placed in Hibernate A medium and kept at 4 °C. For reliable organoid generation, it was imperative that the tissue was processed immediately, as a prolonged time between surgical removal and tissue processing reduced the reliability of GBO generation.

The use of human brain tissue was coordinated by the University of Arkansas for Medical Sciences (UAMS) Tissue Biorepository and Procurement Service (TBAPS) for bio-specimen banking following the ethical and technical guidelines on the use of human samples for biomedical research purposes under the Institutional Review Board-approved protocol (IRB #228443) and general consent form. Patient glioblastoma tissues were collected at the University of Arkansas for Medical Sciences after informed patient consent was obtained, and all patient samples were de-identified before processing. A total of 3 patient cases with the following demographic information (Table 3) were used for this study:

The tissue was transferred to a sterile glass dish with H+GPSA medium containing Hibernate A, 1× GlutaMax (Thermo Fisher Scientific), 1× PenStrep (Thermo Fisher Scientific), and Amphotericin B (Thermo Fisher Scientific) for dissection under an Evos microscope (Thermo Fisher Scientific) within a laminar flow biosafety cabinet. The resected tumors were minced into approximately 0.5 to 1 mm diameter pieces using fine dissection scissors (Fine Science Tools, Foster City, CA, USA) and washed with H+GPSA medium to remove cellular debris. Pieces containing substantial amounts of necrosis or surrounding brain tissue were removed. Tumor pieces were incubated in 1× RBC (red blood cell) lysis buffer (Thermo Fisher Scientific) under gentle rotation for 10 min at room temperature to lyse the majority of contaminating red blood cells. Once the RBC lysis buffer was aspirated, and tumor pieces were washed with H+GPSA medium. 

### 4.6. Generation of GBOs from Resected Patient Glioblastoma Tissue

The tumor pieces were distributed in ultra-low attachment 6-well culture plates (Corning, #CLS3471) with 4 mL of GBO medium containing 50% DMEM:F12 (Thermo Fisher Scientific), 50% Neurobasal medium (Thermo Fisher Scientific), 1× GlutaMax (Thermo Fisher Scientific), 1× NEAAs (Thermo Fisher Scientific), 1× PenStrep (Thermo Fisher Scientific), 1× N2 supplement (Thermo Fisher Scientific), 1× B27 *w/o* vitamin A supplement (Thermo Fisher Scientific), 1× 2-mercaptoethanol (Thermo Fisher Scientific), and 2.5 μg/mL human insulin (Sigma) per well, and placed on an orbital shaker rotating at 120 rpm within a 37 °C, 5% CO_2_, and 90% humidity sterile incubator. Roughly 75% of the medium was changed every 48 h. Within the first week of culture, the tumor pieces often shed cellular and blood debris, making the medium slightly cloudy. The shedding then ceased, and the tumor pieces generally formed rounded organoids within an additional 1-2 weeks, depending on patient-specific tumor growth characteristics. The criteria for the successful establishment of GBOs from a given patient’s tumor was that the micro-dissected tumor pieces survived for 2 weeks, developed spherical morphology, and continuously grew in culture.

### 4.7. GBO Culture for Invasion Assay

After 2 weeks of growth, organoids were placed in an ultra-low attachment round bottom 96-well microplate (Corning, #4515) in 100 uL GBO medium and placed in a 37 °C, 5% CO_2_, and 90% humidity sterile incubator. After cooling the plate for at least 30 min on ice, 100 µL of media was added and the organoids were treated with 10 µM of compound and 33% of Matrigel^®^ (Corning #351234) final concentration.

Migration was observed at 0, 24, 48, 72, 144, 192, 240, and 360 h. using Cytation 5.

## Figures and Tables

**Table 1:** Table Compounds/drugs Panel used for experimentation.

Compound	Simplified Group Identification	Family/Chemical Group	Activity	References
BS-1-28	BS	MMB (Melampomagnolide b) indole esters	NFk-B inhibitors	[27]
BS-2-04	BS	MMB indole esters	NFk-B inhibitors	[27]
BS-4-60	BS	MMB indole esters	NFk-B inhibitors	[27]
BSK-1-97	BSK	MMB-Thiadiazolidinone	NFk-B and GSK-3 B inhibitors	Non-published
BSK-2-17	BSK	MMB-Thiadiazolidinone	NFk-B and GSK-3 B inhibitors	Non-published
BSK-2-26	BSK	MMB-Thiadiazolidinone	NFk-B and GSK-3 B inhibitors	Non-published
JVM-3-55	JVM	MMB dimers	NFk-B inhibitors	[28]
JVM-3-62	JVM	MMB dimers	NFk-B inhibitors	[28]
JVM-4-25	JVM	MMB Triazole dimers	NFk-B inhibitors	[29]
JVM-4-26	JVM	MMB Triazole dimers	NFk-B inhibitors	[29]
PNR-4-44	PNR	Heterocyclic trans-cyanocombretastatin analogues	Tubulin inhibitors	[32]
PNR-4-48	PNR	Heterocyclic trans-cyanocombretastatin analogues	Tubulin inhibitors	[32]
PNR-5-02	PNR	CA-4 triazole	Tubulin inhibitors	[31]
PNR-5-82	PNR	Indole barbiturate and thiobarbiturates	COX-2 inhibitors	[30]
PNR-5-85	PNR	Indole barbiturate and thiobarbiturates	COX-2 inhibitors	[30]
PNR-5-88	PNR	Indole barbiturate and thiobarbiturates	COX-2 inhibitors	[30]
PNR-7-74	PNR	Heterocyclic trans-cyanocombretastatin	Tubulin inhibitors	[32]
PNR-7-84	PNR	Heterocyclic trans-cyanocombretastatin	Tubulin inhibitors	[32]
ST-145	ST	Resveratrol analogues	Tubulin inhibitors	[33]
ST-145(B)	ST	Novel 4,5-disubstituted 2H-1,2,3-triazoles as cis-constrained analogues of combretastatin	Tubulin inhibitors	[34]
ST-148	ST	Resveratrol analogues	Tubulin inhibitors	[33]
ST-467	ST	Novel 4,5-disubstituted 2H-1,2,3-triazoles as cis-constrained analogues of combretastatin	Tubulin inhibitors	[34]

**Table 2:** Summary of compound cytotoxicity and invasion inhibition. Significance is compared to control (DMSO) with ns: non-significant, (-): higher cytotoxicity or death induced than the control, (=): no difference compared to control, ( + ): higher viability of percentage of living cells than the control. Significance was established for cytotoxicity and invasion assay independently with two-way ANOVA with the control (DMSO) for 48 h of invasion, control as the comparative model with the Dunnett model, ns (non -significant) for adjusted *p* > 0.05,* for adjusted *p* ≤ 0.05, ** for adjusted *p* ≤ 0.01, *** *p* ≤ 0.001, and **** *p* ≤ 0.0001. For each experiment, n = 3.

Compound	Activity/Inhibitors	Cytotoxicity	Cytotoxicity Effect in Comparision to Control	Invasion (48 h)	Invasion Inhibition
PNR-5-85	COX-2	*	-	***	Low
BSK-1-97	NFk-B and GSK-3 B	***	-	*	High
JVM-4-26	NFk-B	****	-	**	Moderate
BS-1-28	NFk-B	****	-	*	High
BS-4-60	NFk-B	****	-	**	Moderate
JVM-3-62	NFk-B	*	+	***	Low
PNR-5-02	Tubulin	*	+	**	Moderate
PNR-7-74	Tubulin	*	+	**	Moderate
ST-467	Tubulin	*	+	**	Moderate
JVM-3-55	NFk-B	***	+	**	Moderate
ST-148	Tubulin	***	+	**	Moderate
ST-145	Tubulin	****	+	**	Moderate
ST-145(B)	Tubulin	****	+	ns	Very High
BS-2-04	NFk-B	ns	=	**	Moderate
BSK-2-17	NFk-B and GSK-3 B	ns	=	**	Moderate
BSK-2-26	NFk-B and GSK-3 B	ns	=	**	Moderate
PNR-4-48	Tubulin	ns	=	**	Moderate
PNR-5-88	COX-2	ns	=	**	Moderate
JVM-4-25	NFk-B	ns	=	***	Low
PNR-7-84	Tubulin	ns	=	***	Low
PNR-4-44	Tubulin	ns	=	ns	Very High
PNR-5-82	COX-2	ns	=	ns	Very High

**Table 3:** Tumor/patient demographic information.

Sample ID	Age	Sex	Diagnosis	MGMT Mutation	IDH Mutation
35312	36	Female	Gliosarcoma, rare glioblastoma, primary	no	no
36095	57	Male	Glioblastoma, recurrent	no	no
35456	43	Female	Glioblastoma, primary	yes	yes

## Data Availability

The data presented in this study are available in the manuscript.

## Supplementary Materials

The following are available online at https://www.mdpi.com/article/10.3390/ijms221910720/s1.
