# Supplementary material for: Biobanked Glioblastoma Patient-Derived Organoids as a Precision Medicine Model to Study Inhibition of Invasion
Source: Int J Mol Sci. 2021 Oct 3;22(19):10720. doi: 10.3390/ijms221910720 (PMC8509225; doi:10.3390/ijms221910720)

FigureS1_Compound structures and molecular weights

**Compound**	**MW**	**Compound structure**
**BS-1-28**	407.46	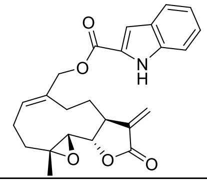
**BS-2-04**	433.49	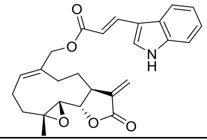
**BS-4-60**	421.48	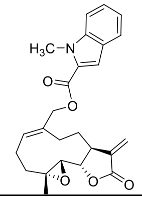
**BSK-1-97**	603.69	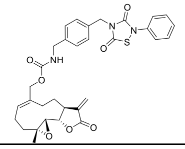
**BSK-2-17**	657.74	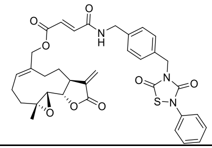
**BSK-2-26**	604.12	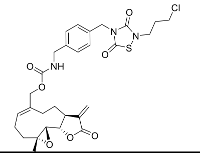
**JVM-3-55**	554.62	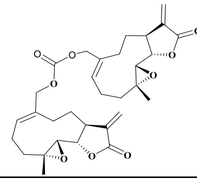
**JVM-3-62**	724.88	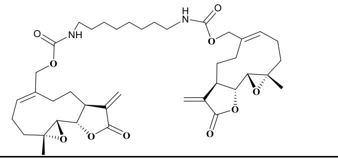
**JVM-4-25**	425.91	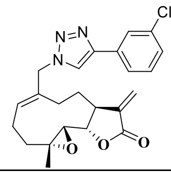

**JVM-4-26**	409.45	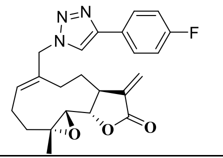
**PNR-4-44**	334.37	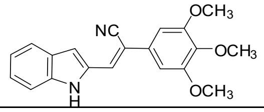
**PNR-4-48**	335.35	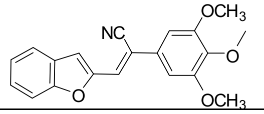
**PNR-5-02**	351.42	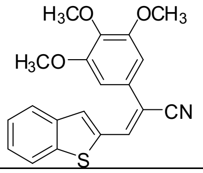
**PNR-5-82**	375.44	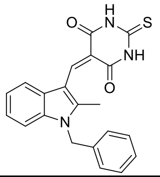
**PNR-5-85**	400.45	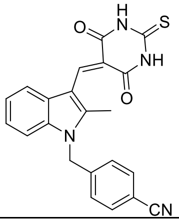
**PNR-5-88**	433.48	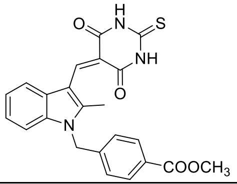
**PNR-7-74**	367.42	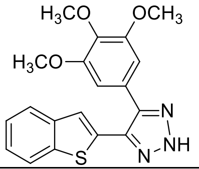
**PNR-7-84**	304.34	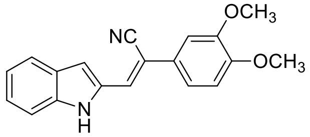
**ST-145**	341.36	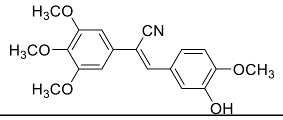

**ST-145(B)**	357.36	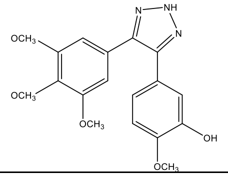
**ST-148**	325.35	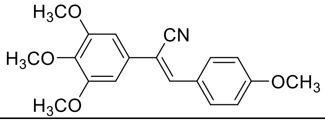
**ST-467**	341.36	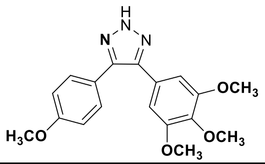

Figure S2_ Standard curve for interpolation of percentage of viability for (a) BS, (c) BSK, (e) JVM, (g) PNR and (i) ST and of live cell number for (b) BS, (d) BSK, (f) JVM, (h) PNR and (j) ST

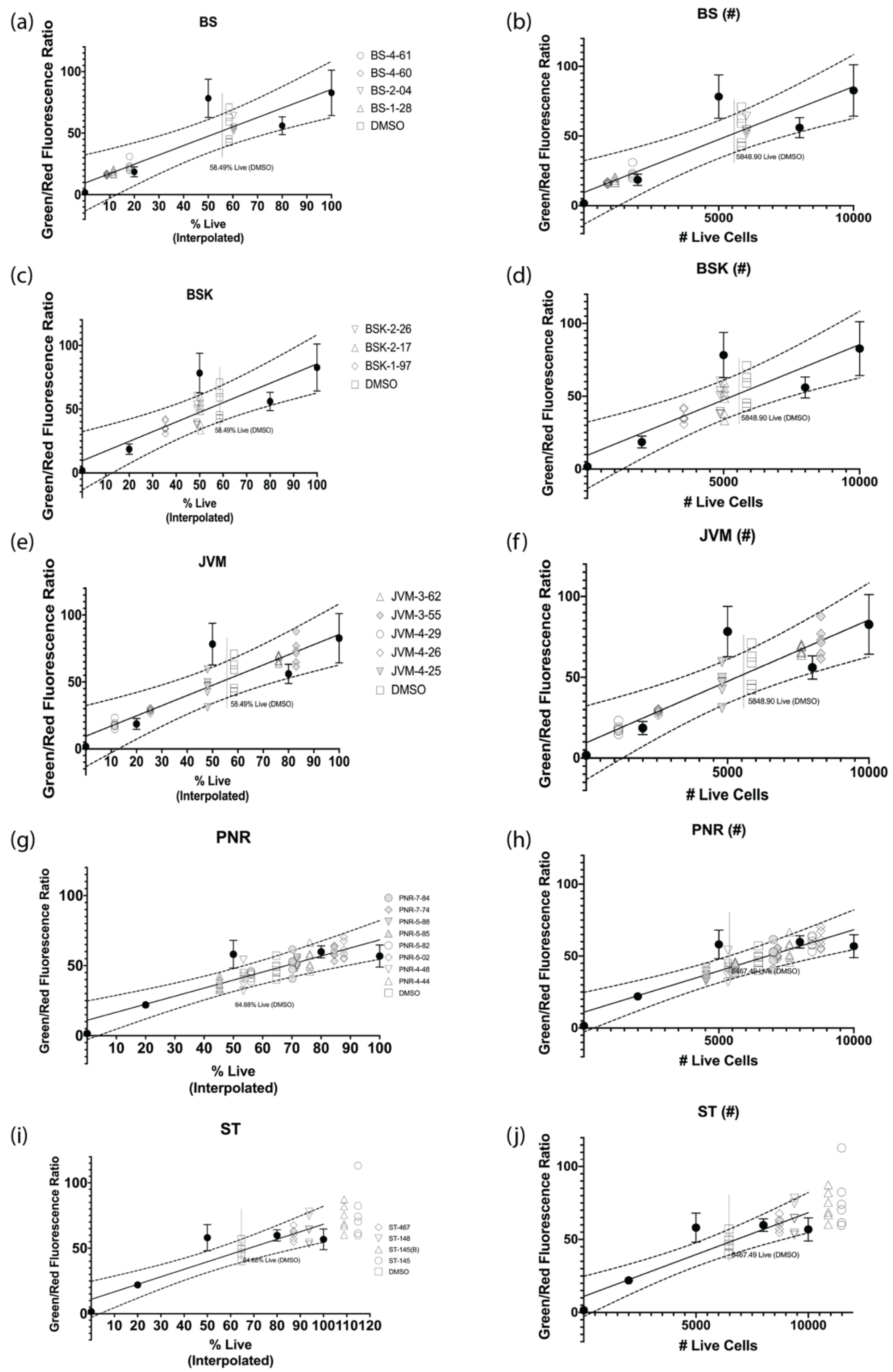

Supplement: Supplementary file 1 [file ijms-22-10720-s001.zip › ijms-1352306-supplementary.pdf]
